# A preliminary investigation of anti-reflux intervention for gastroesophageal reflux related childhood-to-adult persistent asthma

**DOI:** 10.1186/1750-1164-8-3

**Published:** 2014-06-20

**Authors:** Zhi Wei Hu, Zhong Gao Wang, Yu Zhang, Ji Min Wu, Wei Tao Liang, Yue Yang, Shu Rui Tian, Ai E Wang

**Affiliations:** 1 Center for GERD, The Second Artillery General Hospital PLA, Beijing Normal University, No. 16 Xinwai Street, Xicheng district, Beijing, China; 2 Xuanwu Hospital, Capital Medical University, Beijing, China; 3 Department of Respiratory Medicine, Artillery General Hospital, Beijing Normal University, Beijing, China

**Keywords:** Asthma, Gastroesophageal reflux, Stretta radiofrequency, Laparoscopic nissen fundoplication

## Abstract

**Background:**

Childhood-to-adult persistent asthma is usually considered to be an atopic disease. However gastroesophageal reflux may also play an important role in this phenotype of asthma, especially when it is refractory to pulmonary medicine.

**Methods:**

Fifty-seven consecutive GERD patients who had decades of childhood-to-adult persistent asthmatic symptoms refractory to pulmonary medication were enrolled. GERD was assessed by a symptom questionnaire, endoscopy, reflux monitoring, and manometry, and treated by Stretta radiofrequency (SRF) or laparoscopic Nissen fundoplication (LNF). The outcomes were followed up with a questionnaire for an average of 3.3 ± 1.1 years.

**Results:**

Upper esophageal sphincter hypotonia, lower esophageal sphincter (LES) hypotonia, shortened LES, and esophageal body dyskinesia were demonstrated by esophagus manometry in 50.9%, 43.9%, 35.1%, and 45.6% of the patients, respectively. The symptom scores for heartburn, regurgitation, coughing, wheezing, and chest tightness significantly decreased from 5.8 ± 2.0, 5.6 ± 2.0, 7.3 ± 1.6, 8.4 ± 1.2, and 8.1 ± 1.5, to 1.2 ± 1.8, 1.1 ± 1.6, 2.8 ± 2.5, 3.8 ± 2.7, and 3.9 ± 2.7, respectively, after anti-reflux treatment (*P* < 0.001).

**Conclusions:**

Esophagus dysfunction is high in childhood-to-adult persistent asthmatic patients with GERD. SRF and LNF are both effective for esophagus symptoms as well as persistent asthmatic symptoms for these patients. GER may relate with asthmatic symptoms in some patients. Evaluating asthmatic patients for possible treatment of the underlying cause, such as GERD, may improve symptoms and prevent disease persistence.

## Background

Asthma is one of the most common chronic illnesses in children. In the USA, national estimates of current asthma prevalence among the children in selected minority subgroups range from 4.4% in Asian Indian children to 13.0% in American Indian/Alaska Native children [1]. In longitudinal cohort studies, 17%–49% of young children have wheezing with different pediatric asthma phenotypes, of which 4%–14% is a persistent wheeze [2]. Sears et al. repeatedly investigated asthmatic children from 9 to 26 years of age with questionnaires and found that 14.5% had wheezing that persisted from childhood to 26 years of age and 12.4% subsequently relapsed by the age of 26. The factors predicting persistence or relapse were sensitization to house dust mites, airway hyper-responsiveness, female sex, smoking, and early age at onset [3]. Recently, considering asthma as a multi-factorial disease related to familial and environmental influences has become a consensus. The risk factors include family history of asthma, personal history of atopic dermatitis (skin allergies), or allergic rhinitis (such as hay fever) and exposure to air pollutants, especially cigarette smoke [4]. Gastroesophageal reflux disease (GERD) is a common disorder in children that may play an important role in childhood asthma. Although an investigation into a potential association between childhood asthma and GER has not been performed, there are many articles reporting this association in pediatric patients [5]. However, due to methodological limitations of existing studies, the paucity of population-based studies, and a lack of longitudinal studies, several aspects of relationship between GER and asthma in children remain unclear [6]. We have been focusing on treating GERD related respiratory symptoms for 8 years, [7,8] and our fundamental concern is that reflux is a risk factor for recurrent microaspiration, which may play an important role in childhood asthma, including its persistence [9,10]. Therefore, the present study assessed childhood-to-adult persistent asthma with respect to GERD evaluation and active anti-reflux intervention effects in order to evaluate a possible relationship between GER with this asthma phenotype.

## Methods

Clinical data were gathered in a retrospective manner with the approval of the Ethics Committee of The Second Artillery General Hospital. Written informed consent for participation in the study was obtained from all patients.

The patients enrolled in this study met the following criteria: (1) GERD evaluation treated by Stretta radiofrequency (SRF) or laparoscopic Nissen fundoplication (LNF) as described in our previous studies at Center for GERD, The Second Artillery General Hospital PLA, Beijing Normal University, People’s Republic of China;[11,12] (2) >18 years of age; (3) persistent episodic attacks of wheezing and asthma since childhood (1 to 18 years of age), which were identified and diagnosed by physicians in other hospitals for more than 10 years before admission; and (4) had little or insufficient response to long-term and full dose medication for asthmatic symptoms.

### Evaluation of lung function and GER

Basal lung function tests were conducted on admission. The presence of acid GER was tested by ambulatory 24-hour dual pH monitoring, and a De Meester (DMS) score of >14.72 was considered to be positive for increased acid GERD (sensitivity, 96%; specificity, 96%);[13] endoscopic evaluations for hiatus hernia (HH) and esophagitis according to Los Angeles (LA) classification; and esophagus manometry for elucidating hypotonia of the upper esophageal sphincter (UES) and lower esophageal sphincter (LES), length of LES, and esophageal body dyskinesia were all assessed.

### Questionnaire to investigate the symptoms and medication at admission and in the follow-up after SRF or LNF

Questionnaires were completed before the SRF and LNF treatment and then for the follow-up. A 6-point scale ranging from 0 to 5 was applied to assess the severity and frequency of heartburn, regurgitation, coughing, wheezing, and chest tightness according to the Reflux Diagnostic Questionnaire as was applied in our previous studies [14-16]. Medications used for asthmatic symptoms, such as aminophylline, inhaled corticosteroids, inhaled beta-agonists, and oral corticosteroids, amongst others, were documented. Degree of satisfaction (very satisfied, satisfied, acceptable, dissatisfied, and very dissatisfied) toward anti-reflux treatment for asthmatic symptom was investigated at the follow-up.

The outcomes of anti-reflux treatment for asthmatic symptoms were as follows:

(1) Cure: asymptomatic without medication;

(2) Excellent: only occasionally mild, slight, or no asthmatic symptoms. Anti-asthma medication is completely ceased or reduced by more than half;

(3) Good: less than weekly attacks of mild to moderate wheezing. Anti-asthma medication is still in use with various reductions;

(4) Fair: the severity or frequency score is only decreased by one or two points. The patient still has weekly severe or moderate asthmatic symptoms. Drug consumption reduced by less than half or unchanged;

(5) Poor: asthmatic symptoms and drug consumption is unchanged.

### Statistical analysis

Data analysis was performed using SPSS version 13 software (SPSS Inc., Chicago, IL, USA). Comparisons of mean values of the studied parameters before and after treatment were performed using the paired Student’s *t*-test. Comparisons of the magnitude of change for the continuous variables between different groups were carried out using Mann–Whitney *U*-test. Correlations of non-normal variables were assessed using Spearman rank correlation coefficients. All *P*-values less than 0.05 were considered statistically significant.

## Results

This study investigated consecutive asthmatic cases enrolled at our center for GERD from December 2007 to April 2011. In total, 57 cases fulfilled our inclusion criteria and were followed up in January 2013. Among these cases, SRF was carried out in 24 and LNF was performed in 33, with two of the latter having previously received SRF therapy. The mean follow-up duration was 3.3 ± 1.1 years (range 2–6 years). Patient demographics and baseline pulmonary and GER evaluation are summarized in Table 1. The DMS of distal esophagi is more intensive than that of proximal esophagi (*P* < 0.001) and they are correlated (r = 0.268, *P* = 0.018). Patients with HH had higher heartburn score (*P* = 0.036) and weaker LES (*P* = 0.008).

**Table 1:** Patient demographics, baseline pulmonary, and GER evaluation

**Variables**	**Total (n = 57)**
**Sex, M/F**	18/39
**Age (range), years**	47.3 ± 13.3 (20–81)
**Smoker/nonsmoker**	4/53
**Wheezing onset age (range), years**	10.2 ± 4.5 (1–16)
**Duration of wheezing (range), years**	38.1 ± 12.7 (10–70)
**Lung function test**	
FVC, L (% predicted)	3.1 ± 0.9 (93.2 ± 20.6%)
FEV1, L (% predicted)	1.67 ± 0.7 (62.8 ± 21.8%)
FEF, L/sec (% predicted)	4.5 ± 2.2 (66.9 ± 26.3%)
FEV1/FVC	54.6 ± 14.9%
FEV1/FVC < 70%, 30%–49%, < 30%	17 (29.8%), 13 (22.8%), 1 (1.8%)
**Blood eosinophil count, cells/mm**^**3**^**(P%)**	305.2 ± 325.3 (17.5%)
**Esophageal endoscopy**	
Esophagitis (P%)	27 (47.4%)
LA-A, LA-B, LA-C, LA-D	14, 10, 2, 1
Barrett esophagus (P%)	1 (1.8%)
Hiatal hernia (P%)	20 (35.1%)
**Dual 24 hour pH monitoring**	
Distal channel DMS (P%)	41.94 ± 59.11 (64.9%)
Proximal channel DMS (rang)	8.12 ± 10.23 (0.20 - 50.77)
**High-resolution manometry**	
MUESP, mmHg (P%)	41.2 ± 22.7 (50.9%)
MLESP, mmHg (P%)	14.2 ± 6.9 (43.9%)
LHPZ, cm (P%)	2.9 ± 0.7 (35.1%)
Esophageal dyskinesia	26 (45.6%)

*LA*, Los Angeles classification; *DMS*, DeMeester score; *MUESP*, mean upper esophageal sphincter pressure (Normal range: 34–104 mmHg); *MLESP*, mean lower esophageal sphincter pressure (Normal range: 13–43 mmHg); *LHPZ*, length of high pressure zone (Normal range: 2.7–4.8 cm); P%: percentage of positive finding.

**Figure 1:**
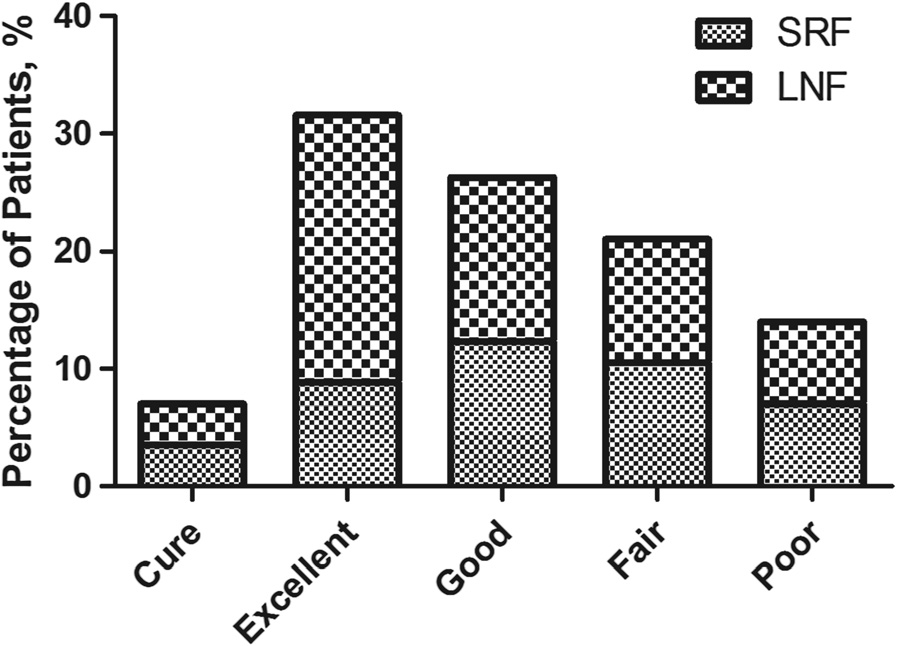
**Overall clinical response of asthmatic symptoms to anti-reflux therapy.** By the end of an average of 3.3 year follow-up, cure, excellent, good, fair, and poor outcomes in the overall asthma status were obtained in 7.0%, 31.6%, 26.3%, 21.1%, and 14.0% of the patients, respectively.

The length of stay after anti-reflux intervention ranged from 2 to 7 days for LNF with a median of 4.0 days, and from 2 to 6 days for SRF with a median of 3.2 days. By the end of an average of 3.3 years of follow-up, the symptom scores for heartburn, regurgitation, coughing, wheezing, and chest tightness significantly decreased from 5.8 ± 2.0, 5.6 ± 2.0, 7.3 ± 1.6, 8.4 ± 1.2, and 8.1 ± 1.5 to 1.2 ± 1.8, 1.1 ± 1.6, 2.8 ± 2.5, 3.8 ± 2.7, and 3.9 ± 2.7 respectively, with reduction rates of 75.1% ± 35.9%, 75.3% ± 35.8%, 58.4% ± 34.9%, 53.9% ± 32.4%, and 51.9% ± 32.7%, respectively (*P* < 0.001; Table 2). Cure, excellent, and good outcome for overall asthma status were obtained in 7.0%, 31.6%, and 26.3% of the patients, respectively, while 21.1% and 14.0% of the patients had fair and poor response to the anti-reflux treatment, respectively (Figure 1). The two patients previously treated by SRF without a satisfactory response later accepted LNF, and one of them was cured and the other had an excellent outcome. In 34 patients who had frequent or daily nocturnal awakening, cough, and wheezing before anti-reflux treatment, 11 (32.4%) patients resumed normal sleeping, while 14 (41.2%) patients markedly improved and their nocturnal episodes became occasional.

**Table 2:** Outcome of anti-reflux therapy over 3.3 ± 1.1 years with respect to esophagus and asthmatic symptoms

**Symptom**	**Number of patents (percent)**	**Symptom score**		**Symptom score reduction rate,%**	***P*-value**
		**Pre-treatment**	**Post-treatment**		**(2-tailed)**
Regurgitation	50 (87.7%)	5.8 ± 2.0	1.2 ± 1.8	75.1 ± 35.9	<0.001
Heartburn	49 (86.0%)	5.6 ± 2.0	1.1 ± 1.6	75.3 ± 35.8	<0.001
***Esophagus symptom***	***50 (87.7%)***	11.3 ± 4.0	2.3 ± 3.2	75.5 ± 35.6	***<0.001***
Cough	46 (80.7%)	7.3 ± 1.6	2.8 ± 2.5	58.4 ± 34.9	<0.001
wheezing	57 (100%)	8.4 ± 1.2	3.8 ± 2.7	53.9 ± 32.4	<0.001
Chest tightness	55 (96.5%)	8.1 ± 1.5	3.9 ± 2.7	51.9 ± 32.7	<0.001
***Asthmatic symptom***	***57 (100%)***	***22.1 ± 5.0***	***9.9 ± 7.2***	***54.3 ± 31.9***	***<0.001***

Moreover, 28.1% of the patients reported that their asthmatic symptoms were getting better, 49.1% of the patients were stable, and 22.8% of the patients responded well to the anti-reflux treatment for 6 months to 2 years before they experienced various degrees of recurrence (Table 3).

**Table 3:** Evolvement of patient clinical state during follow up

	**SRF (n = 24)**	**LNF (n = 33)**	**Total (n = 57)**
Improving	5 (20.8%)	11 (33.3%)	16 (28.1%)
Stable	13 (54.2%)	15 (45.5%)	28 (49.1%)
Partial recurrence	3 (12.5%)	3 (9.1%)	6 (10.5%)
Complete recurrence	3 (12.5%)	4 (12.1%)	7 (12.3%)

In total, 21.1% of patients felt very satisfied and 43.9% felt satisfied about the outcome of their anti-reflux treatment, while 26.3%, 7%, and 1.8% felt acceptable, dissatisfied, and very dissatisfied, respectively (Table 4).

**Table 4:** Satisfactory evaluation of patients with anti-reflux treatment for asthmatic symptoms

	**SRF (n = 24)**	**LNF (n = 33)**	**Total (n = 57)**
Very satisfied	5 (20.8%)	7 (21.2%)	12(21.1%)
Satisfied	8 (33.3%)	17 (51.5%)	25 (43.9%)
Acceptable	7 (29.2%)	8 (24.2%)	15 (26.3%)
Dissatisfied	3 (12.5%)	1(3%)	4 (7%)
Very dissatisfied	1 (4.2%)	0 (0%)	1 (1.8%)

**Figure 2:**
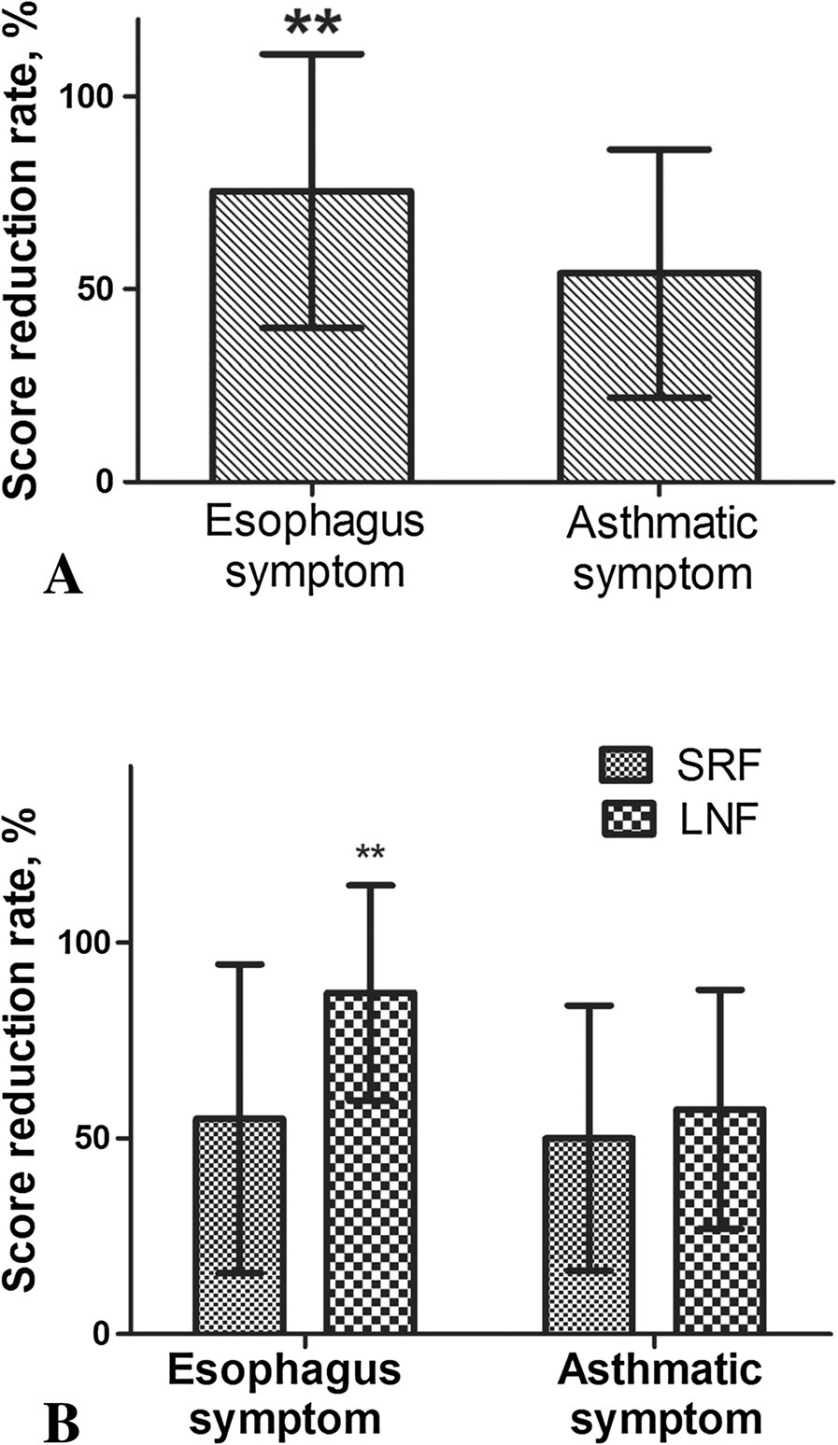
**Effect of anti-reflux treatment on the mean esophagus symptom score and asthmatic symptom score. A**: Esophagus symptom score had significantly better reduction rate than asthmatic symptoms. **B**: The outcome of LNF was significantly better than SRF for esophagus symptoms, but the outcomes of asthmatic symptoms between LNF and SRF groups showed no significant difference. **P < 0.005.

Esophagus symptom scores had significantly greater reductions than asthmatic symptoms (*P* < 0.001; Figure 2A). Compared to SRF, the LNF outcomes were significantly better with respect to esophagus symptoms (*P* = 0.002), but not significant better for asthmatic symptoms (*P* = 0.387; Figure 2B). The reduction rate in asthmatic symptom score was significantly correlated with esophagus symptom (r_s_ = 0.509, *P* < 0.001).

No major complications or deaths occurred during our study. Among patients receiving SRF treatment, some patients experienced several short term complications which disappeared within 1 week, such as throat discomfort in four cases, retrosternal discomfort in six cases, transient nausea/vomiting in three cases, and short-term dysphagia in three cases. As for patients receiving LNF, seven cases had mild dysphagia: three recovered within 1 month; four still had occasional symptoms when eating quickly or solid foods; three cases had prolonged bloating and two cases had increased passage of gas by anus; and three cases had reduced appetite.

## Discussion

The association between asthma and GER has been debated for decades after Sir William Osler first observed the association between worsening asthma and distended stomachs in 1892 [17]. The frequency of GER in asthmatic children was higher than its frequency in other children, which serves as important evidence that asthma and GER are closely related. Historically, the prevalence of GERD in children with asthma ranges from 19.3% to 80.0% with an average of 22.8%. The average rate of abnormal esophageal pH is 68.2% and esophagitis is 35.6% [6] and aspiration of gastric contents in the respiratory tree is not rare in patients with GERD [18]. Theoretically, GER and asthmatic symptoms may be connected through different mechanisms, including micro-aspiration, and both local and central reflexes. In our study, dual pH monitoring identified 64.9% of patients with pathological acid reflux; esophagus endoscopy showed 47.4% of patients had esophagitis.

Our study also found a high prevalence of disorders that may increase GER by endoscopy and HRM. For instance, HH was found in 35.1% of the patients by endoscopy. Furthermore, UES hypotonia, LES hypotonia, shortened LES, and esophageal body dyskinesia were demonstrated by esophagus manometry in 50.9%, 43.9%, 35.1%, and 45.6% of the patients, respectively (Table 1). Although neither HH nor weakened LES showed significantly higher DMS in this study, HH or LES hypotonia often predicts a higher risk of GERD due to its negative impact on esophagus [19,20]. Although 24 hour pH-monitoring is one of the current reference-standard methods for GER assessment in children, it only detects acid reflux. A multichannel intraluminal impedance and pH (MII-pH) monitoring, which detect anterograde or retrograde acid or non-acid bolus and determine the composition, might be more sensitive for GER [21]. These aforementioned modalities may have important diagnostic and therapeutic implications for children or adults with difficult to control or consistent asthmatic symptom. Recently, Negro et al. reported that esophageal acidification has a good level of both sensitivity and specificity by enhancing the Methacholine response in forced expiratory volume in 1 second (FEV1) only in the presence of acid GERD. This test could be a potentially useful tool for better selection of GER-related asthma in clinical practice [22].

Nearly 50% of children have wheezing with respiratory illnesses in their first year of life, and 20% will have continued wheezing in later childhood, which predicts a probability of asthma [23]. The natural history of GER in humans may provide clues to this phenomenon. For instance, in the first 3 months of life, postprandial reflux is considered a physiological event that gradually decreases and disappears by 1 year of age [24]. The progressive decrease in episodes is due to maturation of the LES and acquisition of sitting and standing. However, some children have persistent regurgitation or reflux after the age of one; their reflux is not only associated with feeding, backwardness, irritability, unjustified crying, sudden waking, persistent esophageal hiatus defect, and esophagus malfunction, but also with the strenuous asthmatic symptoms [25,26].

GER-related cough is defined as a cough that is improved or resolved by GER therapy, thus analogically GER-related asthmatic could also be defined by anti-reflux outcome. Our fundamental concern for GER-related asthmatic patients is that reflux is a risk factor for recurrent microaspiration and irritation; therefore, effective GER control is essential for the management of GER-related respiratory symptoms [9]. Anti-acid therapies, such as proton pump inhibitors (PPI), have been tested in randomized trials for asthmatic children and adults and the results revealed that PPI has no clear benefit on asthma control compared to placebo [27,28]. Furthermore, surgical therapies in different uncontrolled series of children with severe persistent asthma have also been reported and most of the selected patients experienced various responses [29-31]. Studies of the outcomes of surgical treatment may be less valuable in evidence based medicine as they suffer from a lack of controls and blinding, use different postoperative evaluation criteria, and are typically based on a highly selective group of patients. However, in our opinion, restoring the anatomical barrier of gastroesophageal junction, by SRF or LNF, reduces the volume, frequency, duration, and/or destination of GER so that the related aspiration and irritation resolution may be superior to medication therapy, such as PPI which mainly reduces the acidity of GER and is of high recurrence when off medication. Thus, surgery may have better value in the management of reflux-related respiratory symptoms as presented in this study and previous LNF and SRF studies [11,12,14,32].

As shown in the present study, 64.9% of the patients had good or better response to SRF or LNF therapy, while 35.1% of the patients still had less than good outcomes and 22.8% of the patients experienced different degrees of recurrence. This data suggests that the anti-reflux therapy at hand is not a panacea in these selected asthmatics as a whole. It is still difficult to define whether an a priori subset of asthmatics will improve with anti-reflux therapy. Complications, such as mild dysphagia and bloating, were evident in patients after receiving LNF; for instance, five of the seven dysphagia patents and two of the three bloating patients were found to have esophageal dyskinesia by esophagus manometry, which indicates that pre-LNF esophageal dyskinesia may represent a risk factor for complications after LNF. In this study, we showed that the outcome of asthmatic symptoms is highly related to the effectiveness of anti-reflux therapy and that their symptom scores are significantly correlated. The two patients who failed on SRF treatment in this study still had a second chance to attain a successful outcome when effective LNF is applied. Therefore, effective GER control is one of the key factors for successful GER-related asthmatic management. Although SRF treatment is less effective for esophagus symptoms, it is relatively less invasive with fewer complications. Furthermore, there has not been a perfect anti-reflux approach that can completely bring GER under control while avoiding complications and recurrence. Thus, it is still valuable to offer re-intervention for patients who failed on primary anti-reflux treatment.

Another key element for GER-related asthma is persistent airway hyperactivity. In the present study, although remarkable outcomes were obtained after effective GFR control, the majority of patients still reported exacerbations of asthmatic symptoms, which could be triggered by catching a cold, exertion, and/or strong odors, such as cigarette smoke and petrol fumes. Therefore, systemic corticosteroids were discontinued after SRF or LNF, while other anti-asthma medications, such as inhaled corticosteroids, inhaled beta-agonists, and/or aminophylline with reduced dosage, were still maintained in most patients.

Although the majority of asthma patients may obtain the targeted level of control with pulmonary medication, some patients will not achieve control even with the best therapy, [33] a group which probably accounts for less than 5% of all children with asthma. The management of this group of children is complex, with little evidence to guide the choice of further treatment for those who remain symptomatic even after the use of regular systemic corticosteroids. The lives of children with difficult to treat asthma are severely disrupted with frequent hospital visits, school absence, and limitations in normal activities. Behavioral problems and a lower quality of life are more pronounced in those children [34]. However, a review by Mutius et al. [35] indicates that over the past two centuries real progress has been made in asthma research. Allergic responses that often substantially contribute to both chronic persistent asthma and acute exacerbations of asthma symptoms have been extensively studied and may be overemphasized. The precise nature of this inflammation remains a mystery, the fundamental causes of asthma are still not understood, and asthma still has no cure. The medications at hand provide symptomatic relief and improve lung function and airway responsiveness, but they do not prevent exacerbations or disease progression. However, studying active anti-reflux in GER-related asthmatics may partly provide alternative insights into mechanisms and management of this disorder.

## Conclusions

Esophagus related mortality is high in childhood-to-adult persistent asthmatic patients with GERD. SRF and LNF are both effective for treating esophagus symptoms as well as GERD-related persistent asthmatic symptoms. GER may also play an important role in this asthma phenotype. The evaluation of patients for possible treatment of the underlying causes of asthma, such as GER, can improve symptoms and prevent disease persistence. However, large-scale and controlled studies are further indicated.

## Abbreviations

GER: Gastroesophageal reflux; SRF: Stretta frequency; LNF: Laparoscopic Nissen fundoplication; CT: Computed tomography; HRM: High-resolution manometry; DMS: DeMeester score; HH: Hiatal hernia; LES: Lower esophageal sphincter; PPI: Proton pump inhibitors; FVC: Forced vital capacity; FEV1: Forced expiratory volume in 1 second; FEF: Forced expiratory flow; LA: Los Angeles classification; MUESP: Mean upper esophageal sphincter pressure; MLESP: Mean lower esophageal sphincter pressure; LHPZ: Length of high pressure zone.

## Competing interests

The authors declare that they have no competing interests.

## Authors’ contributions

ZWH studied and analyzed the data, conducted literature reviews, and drafted the manuscript. ZGW designed the study and helped draft the manuscript. ZWH, YZ, JMW, WTL, YY, SRT and AEW carried out the study, collected the data, and helped draft the manuscript. All authors read and approved the final manuscript.

## Authors’ information

ZW Hu and SR Tian the attending doctor, Y Zhang, WT Liang and Y Yang the resident of Center for GERD, AE Wang the attending doctor in Department of Respiratory Medicine, The Second Artillery General Hospital.

ZG Wang was a so called severe asthma patient with severe and intolerable respiratory symptoms requiring constant medication for years. After GER was confirmed and fundoplication performed, his asthma disappeared completely without taking any medication at present [7]. He then decided to devote himself to rescue patients who suffered as he did; therefore, The GER Center for related airway disorder was established in 2006. He is also the pioneer of GER related airway disease research and practice in the People’s Republic of China, the founder of the Center for GER of Second Artillery General Hospital, Professor and Director of Vascular Institute of Xuanwu Hospital of Capital Medical University Capital, Life Long President of the Chinese Vascular Society, and Vice President of International Society of Vascular Surgery.

JM Wu is the director and chief physician of the Center for GERD of Second Artillery General Hospital.
